# Treatments for Non-Small-Cell Lung Cancer: The Multiple Options for Precision Medicine

**DOI:** 10.3390/curroncol29100558

**Published:** 2022-09-28

**Authors:** Elvire Pons-Tostivint, Jaafar Bennouna

**Affiliations:** 1 Medical Oncology, Centre Hospitalier Universitaire Nantes, Nantes University, F-44000 Nantes, France; 2 Department of Medical Oncology, Hospital Foch, F-92150 Suresnes, France

In recent years, advances in molecular diagnostics have transformed the management of advanced non-small-cell lung cancer (NSCLC), allowing for increasingly personalized approaches. As molecular testing strategies have evolved, new potentially actionable molecular alterations have been discovered, enabling treatment with new targeted therapies. Immunotherapy has been added for the treatment of lung cancer after surgery, radiotherapy, chemotherapy, and targeted therapy, in all stages of the disease. The management of locally advanced tumors has become highly complex, and improving the early detection of lung lesions has become more important than ever with the improvement of therapies. In this Special Issue, entitled “Treatments for Non-Small Cell Lung Cancer: The Multiple Options for Precision Medicine”, our goal is to provide an overview of advances in lung cancer management at different disease stages. 

While the number of actionable molecular alterations is growing, current guidelines recommend comprehensive genotyping approaches such as next-generation sequencing (NGS) over sequential single-gene testing. However, there is still a lack of consensus regarding the ideal balance of thoroughness and timeliness. In the context of first-line therapy, a workflow solely based on NGS is time-consuming in the detection of EGFR mutations. Alternative techniques have been developed, such as the IdyllaTM EGFR mutation assay, i.e., a fully automated real-time polymerase chain reaction detecting EGFR mutations with minimal delays. Petiteau et al. [1] report results of a prospective study that assessed whether the early genotyping of EGFR can enable the early initiation of efficient treatment with tyrosine kinase inhibitors (TKIs). In total, 223 patients were prospectively tested with both reference methods, NGS and IdyllaTM EGFR mutation assays, to determine whether rapid EGFR genotyping tests could guide earlier therapy decisions. This paper discusses the relevance for integrating EGFR-focused assays into the parallel NGS testing workflow to shorten the time to treatment. 

Anaplastic lymphoma kinase (ALK) gene rearrangements are found in approximately 3–5% of NSCLC advanced disease. During the past 10 years, multiple ALK inhibitors have been developed. A case report of alkaline phosphatase (ALP) elevation over 6N after the initiation of alectinib, in a patient with a rapid tumor shrinkage, is discussed here. The authors raised the question of whether the early occurrence of the exacerbation of the ALP was a TKI-related toxicity, or whether it was predictive of a tumor response to alectinib. More insights are still needed regarding predictive biomarkers of response and/or resistance to TKIs in this population.

Another alteration of key importance is ROS-1, a rearrangement found in 0.9–2.6% of NSCLCs. ROS-1 plays a major role in the activation of several signaling pathways associated with differentiation, proliferation, cell growth, and survival. ROS-1 rearrangement causes kinase activity deregulation of the protein and the abnormal activation of signaling pathways. Two TKIs have been validated as first-line therapy so far: crizotinib and entrectinib. This present review by Gendarme et al. [2] describes the current knowledge of ROS-1 rearrangement in NSCLCs, including their diagnostic modalities, epidemiology, and characteristics; the development of diverse molecules targeting ROS-1 and the identified resistance mechanisms.

Another set of articles in this issue discuss the optimal management of patients with locally advanced disease. The first article by Drevet et al. [3] focuses on tumors invading the cervical spine, whereas the work conducted by Martel-Lafay et al. [4] deals with the management of unresectable stage III patients with peripheral primary tumors. Drevet et al. [3] report a monocentric retrospective study evaluating two surgical techniques in this population. For these patients with invasion of the cervical spine, multimodal treatments have demonstrated considerable improvements in survival, and the use of induction chemoradiotherapy combined with radical en bloc resection should be used to achieve complete resection. Technical aspects of surgical resection have evolved over the past few years and are discussed in this publication. The authors compare two surgical techniques: a one-step surgical lung and vertebral resection during the same operating time, versus a two-step surgery, i.e., vertebral posterior osteotomy and spine stabilization first, and secondly, an anterior, or posterolateral approach to complete vertebral resection with en bloc lung and parietal resection. They assessed the postoperative morbidity, mortality, and long-term survival of both approaches.

The study by Martel-Lafay et al. [4] deals with the complex management of unresectable stage III NSCLC patients with peripheral primary tumors. When this study began, concomitant radio–chemotherapy (RT-CT) was the recommended treatment for unresectable stage III NSCLC (RT 2 Gy/d, 5 d/week, up to 60 Gy). In this prospective, single-stage, multicenter phase II trial, patients with peripheral primary tumors underwent stereotactic body radiation therapy (SBRT) to the primary tumor after conventional RT-CT for hilar or mediastinal nodal involvement. A peripheral tumor was defined as a tumor located 2 cm or more from the mediastinal organs. RT delivered 66 Gy on mediastinal and hilar lymph node involvement without treating the peripheral tumor. SBRT for the primary peripheral tumor started within 3–4 weeks after the end of RT-CT and delivered 54 Gy in 3 fractions. The primary endpoint was the local control rate at 6 months. Even if this study was stopped early before completion due to the authorization of Durvalumab after RT-CT, data on feasibility and safety are of interest in the use of SBRT in stage III NSCLC in this subset of patients with peripheral tumors. More investigations are now needed in this era of adjuvant immunotherapy.

Although major progress has been made in the treatment for advanced-stage NSCLC patients in the last decade, far less has been made in localized stages. To transfer this progress to earlier-stage disease, much more clinical and translational research efforts are needed. Surrogate markers of overall survival in a neo-adjuvant setting need to be defined, because the paradigm is expected to be drastically changed by neo-adjuvant immunotherapy alone or in combination in the near future. In their review, Chen et al. [5] discuss which indicator between the rate of complete resection, the objective response rate, the major pathological response (MPR), and the pathological complete response (pCR) has a more predictive value for overall survival, considering different neo-adjuvant therapies (chemotherapies, targeted therapies, or immunotherapies). The authors discuss the importance of discrepancies between radiographic response and pathological response, considering that the traditional RECIST criteria cannot accurately appraise the efficacy of immune-related therapy. As more trials use MPR and pCR as surrogates of clinical benefit from neo-adjuvant therapies, it is urgently needed to demonstrate that these pathologic end points capture the magnitude of survival benefit.

The last paper describes key challenges in obtaining an adequate sample in patients with lung nodules. It remains unclear how often a cytologic diagnosis of atypia was ultimately found to be a malignancy. The purpose of this study was to evaluate the duration of time and potential risk factors associated with the development of lung cancer among patients with an initial diagnosis of atypia. Findings from this study indicated that 75% of patients (80/106) with an initial diagnosis of atypia were finally diagnosed with cancer. Of those, half were diagnosed within 35 days, and 75% were confirmed to have lung cancer within 6 months. Additionally, PET-positive patients with mixed ground glass opacities > 3.5 cm in size were at a significantly increased risk of developing lung cancer. This study emphasizes that repeated efforts should be made to establish a diagnosis in patients with lung nodules initially classified as atypia on biopsy.
